# A Simplified Method for the Efficient Refolding and Purification of Recombinant Human GM-CSF

**DOI:** 10.1371/journal.pone.0049891

**Published:** 2012-11-14

**Authors:** Christy A. Thomson, Melanie Olson, Linda M. Jackson, John W. Schrader

**Affiliations:** The Biomedical Research Centre, University of British Columbia, Vancouver, British Columbia, Canada; New York University, United States of America

## Abstract

Human granulocyte macrophage colony-stimulating factor (hGM-CSF) is a haematopoietic growth factor and proinflammatory cytokine. Recombinant hGM-CSF is important not only as a research tool but also as a biotherapeutic. However, rhGM-CSF expressed in *E. coli* is known to form inclusion bodies of misfolded, aggregated protein. Refolding and subsequent purification of rhGM-CSF from inclusion bodies is difficult with low yields of bioactive protein being produced. Here we describe a method for the isolation, refolding and purification of bioactive rhGM-CSF from inclusion bodies. The method is straightforward, not requiring extensive experience in protein refolding nor purification and using standard laboratory equipment.

## Introduction

Granulocyte macrophage colony-stimulating factor (GM-CSF) is a cytokine important for the stimulation of proliferation, differentiation and survival of many hemopoietic cells [1] including mature neutrophils, macrophages and dendritic cells [2], [3]. GM-CSF is produced by many cell types within the body (e.g. fibroblasts, endothelial cells) when stimulated by microbial products or inflammatory cytokines and its activity is important to the innate immune response. T lymphocytes also produce it when stimulated with a specific antigen. As well, because of its ability to stimulate hemopoietic cells, recombinant human GM-CSF (rhGM-CSF) is used, for instance, as a biotherapeutic for immunocompromised individuals undergoing chemotherapy [4]. Dysregulation of GM-CSF activity has been implicated in such auto-immune conditions as arthritis and multiple sclerosis [5]. Neutralization of the bioactivity of GM-CSF by auto-antibodies causes another auto-immune disease, idiopathic pulmonary alveolar proteinosis [6] and GM-CSF is used to treat this condition [7], [8].

GM-CSF is a 127 amino acid compact glycoprotein composed of a two-stranded antiparallel β-sheet and a four α-helix bundle [9]. To purify significant amounts of rhGM-CSF, many attempts have been made to express the protein in *E.coli*. However, with many over-expression systems it was found that rhGM-CSF formed inclusion bodies [10], [11]. Inclusion bodies are aggregates of improperly folded intermediates of proteins that are often formed when mammalian proteins are expressed in *E. coli* and are an ongoing issue in biotechnology and biomedical research [12], [13]. Advancements in inclusion body resolubilization and protein refolding have led to a better understanding of the forces governing inclusion body formation and of methods used to efficiently refold proteins of interest [14], [15]. To generate a sufficient amount of purified protein from inclusion bodies it is necessary to isolate the inclusion bodies from the *E. coli* lysate, solubilize the inclusion bodies and then refold the protein of interest [12]. The first two steps are relatively straightforward often requiring mechanical disruption (e.g. French Press or sonication) and centrifugation to lyse the *E. coli* and isolate the inclusion bodies followed by the addition generally of chaotropic agents (e.g. GuHCl or Urea) to solubilize them. It is the refolding of the protein of interest that is often the limiting step with aggregation and improper disulphide bond formation being two major issues to overcome. During protein refolding, aggregation of partially folded intermediates can result in a significant decrease in final yields. L-arginine, a natural amino acid, is commonly used to enhance protein refolding by suppressing protein aggregation [16], [17]. To aid in proper disulphide bond formation, the inclusion bodies are first solubilized in a solution containing a reducing agent such as DTT or 2-mercaptoethanol. These are used to disrupt any non-native disulphide bonds and must be removed, often by dialysis, before proper disulphide bond formation can proceed in the refolding process. During refolding, a combination of reduced and oxidized thiols (e.g. glutathione), are used to promote disulphide exchange [12]. It is thought that this is most effective when the protein is forming its secondary and tertiary structure so that the cysteinyl residues are in the correct proximity to each other [18].

We describe in detail here a straightforward method to refold and purify rhGM-CSF from inclusion bodies that generates milligram amounts of active protein from a single litre of *E. coli*. The refolding protocol described was also successfully used to refold Fab fragments of antibodies and thus may be used as a general refolding strategy for proteins forming inclusion bodies in *E. coli* such as many cytokines [19].

## Materials and Methods

### Cloning of rhGM-CSF into a Expression Construct

The cDNA of rhGM-CSF, minus the signal sequence, was amplified and ligated into the NdeI and XhoI sites of the pET40b(+) vector (Novagen). The primers, sGMCSF1 (5′ AACATATGGCACCCGCCCGCTCG) and asGMCSF1 (5′TTCTCGAGCTCCTGGACTGGCTCC) were used to amplify the cDNA to initiate with the MAPARS protein sequence at the N-terminus and to remove the stop codon at the C-terminus so to allow incorporation of the C-terminal his-tag. The resultant construct contained the additional amino acids, LEHHHHHHHH, C-terminal to the GM-CSF sequence and was termed pET40-GM-CSF.

### Expression of rhGM-CSF in *E.coli*

The pET40-GM-CSF construct was transformed into the BL21(DE3) strain of *E. coli*. A 10 ml culture using LB containing 30 µg/ml of kanamycin was grown overnight shaking at 37°C and used to inoculate 1 L of LB containing kanamycin (in a 2.8 L Fernbach flask) the following morning. Through prior variation of induction times and lengths, it was found that addition of 1 mM isopropyl β-D-1-thiogalatopyranoside (IPTG) at a culture optical density (600 nm) of approximately 0.3 and subsequent culturing for 5 hrs at 37°C yielded the highest expression of rhGM-CSF. Cells were harvested by centrifugation at 5000×g (Sorval GS3 rotor) at 2–8°C for 10 minutes and stored at −20°C.

### Isolation of Inclusion Bodies

Cells were resuspended in 25 ml of lysis buffer (50 mM Tris, pH 8, 2 mM EDTA, 0.5% Triton X-100 with the addition of Complete Mini Protease Inhibitor Tablets (Roche)). Mechanical disruption was used to lyse the cells (i.e. French Press or sonication) and the inclusion bodies isolated from the cell supernatant by centrifugation at 18000×g at 2–8°C for 20 minutes. The inclusion body pellet was solubilized in resuspension buffer (50 mM Tris, pH 8, 6 M GuHCl (Sigma, G4505), 10 mM DTT) by repeatedly passing the inclusion bodies through an 18g syringe. It is worth noting that any insoluble material can be centrifuged out at this time at 18000×g at 2–8°C for 20 minutes. The resuspended protein material was then diluted 50% in dialysis buffer #1 (50 mM Tris, pH 8, 2 M GuHCl) resulting in a 4 M GuHCl containing solution. The protein solution was then dialyzed overnight at 4°C in snakeskin dialysis tubing (Pierce) against 2 L of buffer #1. The following day the dialysis buffer was changed to 2 L of dialysis buffer #2 (50 mM Tris, pH 8, 1 M GuHCl, 0.4 M Arginine (Sigma, A5006), 3 mM Reduced Glutathione, 0.9 mM Oxidized Glutathione) for overnight dialysis at 4°C. The following day the dialysis buffer was diluted 50% with water and dialysis continued overnight. Any insoluble material was centrifuged (18000×g at 2–8°C for 20 minutes) and the remaining protein solution dialyzed overnight at 4°C against 1 L of dialysis buffer #3 (50 mM Tris, pH 8, 250 mM NaCl, 0.1 M Arginine, 3 mM Reduced Glutathione, 0.9 mM Oxidized Glutathione) to remove the remaining GuHCl.

### rhGM-CSF Purification

The final dialyzed protein solution was clarified by centrifugation (18000×g at 2–8°C for 20 minutes) and the supernatant loaded onto a ProBond (Invitrogen) His-tag affinity column equilibrated in equilibration buffer (50 mM Tris, pH 8, 500 mM NaCl). The column was washed with an excess of wash buffer (50 mM Tris, pH 8, 1 M NaCl, 0.1% Triton X-100) followed by a wash with equilibration buffer to remove the Triton X-100. The rhGM-CSF was eluted in 1 ml aliquots from the resin using elution buffer (50 mM Tris, pH 8, 250 mM Immidazole). To quickly visualize the eluted protein, 10 µl from individual fractions was pipetted on Whatmann filter paper, and stained with Coomassie Brilliant Blue for 2 min before destaining or analyzed by 15% reducing SDS PAGE. Fractions containing eluted protein were pooled and dialyzed against 2 L of 20 mM HEPES, pH 7.8, overnight at 4°C. Protein that precipitated out during the overnight low salt dialysis was removed by centrifugation before proceeding. The purified rhGM-CSF was quantified by UV spectroscopy using a calculated molar absorption coefficient corresponding to the non-reduced rhGM-CSF sequence (e_280nm_ = 14 238) [20]. With the additional LEHHHHHHHH at the C-terminus and resulting higher molecular weight, the absorbance value (A^0.1%^) of the folded rhGM-CSF at 280 nm is 0.89 compared to 0.98 of rhGM-CSF minus the additional amino acids.

### Mass Spectrometry

The purified rhGM-CSF was digested individually with trypsin or protease V8 and the generated peptides separated and analysed by LC-MS/MS (FT-ICR). The peptide mixtures were separated on a PicoTip column (o.d. = 360, I,d. = 75, tip = 15±1 µm) from New Objective (Woburn, MA, USA) packed with reverse-phase C18 material (15 cm, C18 magic, 100 Å, 3 µm, Michrom Bioresources, Auburn, CA, USA). Solvent A (0.5% acetic acid) and solvent B (80% acetonitrile +0.5% acetic acid) were employed. A linear gradient of 6% to 80% solvent B in 30 min at a flow rate of 600 nl/min was applied via an Agilent 1100 nano HPLC pump. Peptide sequences were identified by searching spectra against the Swiss-Prot database using MASCOT [21].

### Bioactivity Assay

The rhGM-CSF bioactivity was analyzed with a proliferation assay using TF-1 cells [22].

The TF-1 cells (ATCC number CRL-2003) were maintained in RPMI medium supplemented with 10% fetal bovine serum (FBS), L-glutamine, sodium pyruvate, and penicillin/streptomycin (all from Gibco) with 2% gibbon IL-3 added just prior to use. The cells were grown in a 37°C humidified incubator with 5% CO_2_. Commercial rhGM-CSF was obtained from ImmunoTools (cat # 11473127, lyophilized), reconstituted, aliquoted and stored at −80°C. For the proliferation assay, TF-1 cells were washed 5 times in RPMI with no supplements and seeded 1000 cells per well in growth medium with no IL-3 or rhGM-CSF in 96 well tissue culture plates (BD Falcon # 353072). rhGM-CSF was serially diluted two-fold in growth medium and added to the washed cells to give final concentrations ranging from 12.8 ng/mL to 1.56 pg/mL, and 100 µL total volume per well. Control wells containing cells but no rhGM-CSF (blank) and rhGM-CSF with no cells were performed. The plates were incubated in a 37°C humidified incubator with 5% CO_2_ for 4 days. Cell Proliferation Reagent WST-1 (Roche cat # 11 644 807 001) was added and incubation continued as above for 4 hrs. The absorbance values at wavelength 450 nm, with reference wavelength of 690 nm values subtracted, were determined using a plate reader (Molecular Devices). The absorbance values were directly proportional to the number of viable cells because the tetrazolium salts in the WST-1 reagent were cleaved to formazan by mitochondrial dehydrogenases in the cells. The blank values were subtracted from all wells on each plate and the values plotted. Unit values were defined as 50 U/mL being equivalent to the concentration of GM-CSF that supports 50% of maximal growth under the assay conditions used.

## Results

### rhGM-CSF Expression and Purification

**Figure 1:**
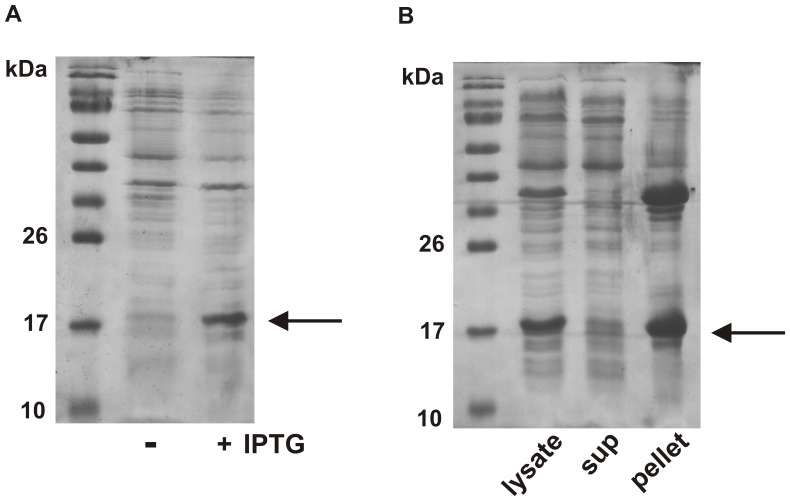
rhGM-CSF forms inclusion bodies when expressed in *E. coli*. (A) Shown is the 15% reducing SDS PAGE analysis of the expression of rhGM-CSF transformed into the BL21(DE3) *E. coli* strain. Upon addition of IPTG (+) the cells efficiently express the rhGM-CSF protein. (B) Following induction with IPTG, the bacteria were lysed by mechanical disruption and the cell supernatant separated from the pellet by centrifugation. Shown is the 15% reducing SDS PAGE analysis of the cell lysate prior to centrifugation, and the subsequent centrifugal supernatant and pellet fractions. The molecular weight marker is PageRuler Prestained Protein Ladder from Fermentas Life Sciences and the gels are stained with Coomassie Brilliant Blue.

The rhGM-CSF protein was efficiently expressed using the pET40b(+) vector in *E. coli* (Fig. 1A). However, the overexpression resulted in the formation of inclusion bodies as the rhGM-CSF was only found in the insoluble pellet after cell-lysis and not in the soluble fraction (Fig. 1B). Lysis of the cells and removal of the supernantant is a first step in the purification of recombinant proteins as many of the endogenous *E. coli* proteins are either soluble or removed from the membrane cellular debris by the detergent used in the lysis buffer. If a means of mechanical disruption of the *E.coli* (i.e. sonicator or French Press) is not available, it is possible to proceed directly to the resuspension step. There will simply be more contaminating proteins in the dialysis tubing and more extensive washing of the purification column may be required.

**Figure 2:**
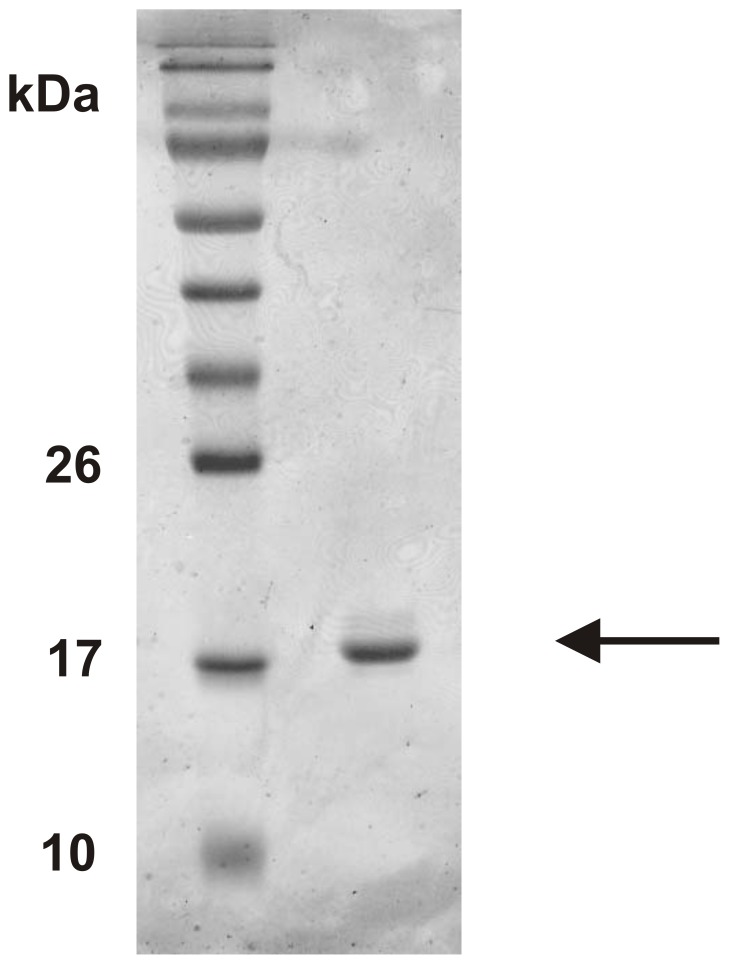
rhGM-CSF can be purified following refolding from inclusion bodies and His-tag affinity chromatography. Shown is the 15% reducing SDS PAGE analysis of purified rhGM-CSF following His-tag affinity chromatography and low salt dialysis. The molecular weight marker is PageRuler Prestained Protein Ladder from Fermentas Life Sciences and the gel is stained with Coomassie Brilliant Blue.

After centrifugation of the cell-lysate, the pelleted inclusion bodies were first solubilized in a buffer containing 6 M GuHCl and 10 mM DTT and then slowly dialyzed against buffers containing decreasing amounts of GuHCl, as well as L-arginine and the redox pair of oxidized and reduced glutathione. This process efficiently refolds the rhGM-CSF and any unfolded/insoluble material is removed by a centrifugation step. The solubilized rhGM-CSF was subsequently purified using a nickel-chelating resin via the 8×His tag engineered on its C-terminus (Fig. 2). From a 1 L culture, approximately 7 mg of soluble and purified rhGM-CSF can be routinely purified.

### Mass Spectral Analysis

**Figure 3:**
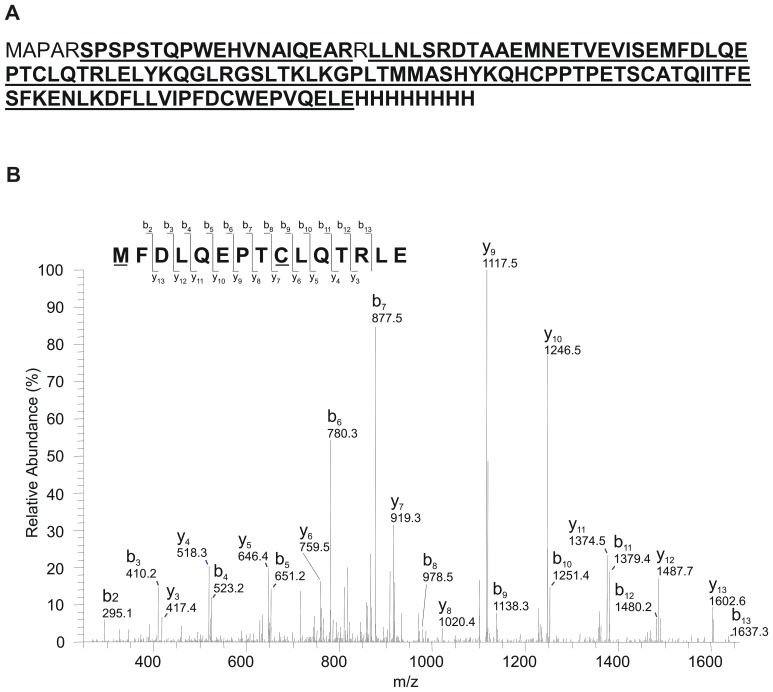
Mass spectral analysis of purified rhGM-CSF. (A) Shown is the sequence of the expressed rhGM-CSF. In bold and underlined is rhGM-CSF sequence coverage obtained by the mass spectral analysis. 95% sequence coverage was obtained, not including the 8×His tag. The rhGM-CSF was cleaved separately by trypsin and Protease V8 and the generated peptides likewise analyzed separately by LC-MS/MS (FT-ICR). (B) The MS/MS spectrum of the doubly charged peptide **MFDLQEPTCLQTRLE** (m/z 948.95), which is the fragment of protein granulocyte-macrophage colony-stimulating factor (amino acid residues 63–77) digested by protease V8, is shown as a representative spectra. **M**: Methionine oxidation; **C**: Cystine carbamidomethyl modification.

To confirm the identity of the purified rhGM-CSF, LC-MS/MS (FT-ICR) was performed on trypsin and protease V8-digested fragments. Trypsin cleaves C-terminal to arginine and lysine residues whereas protease V8 cleaves C-terminal to glutamic and aspartic acids. The mass spectral analysis confirmed the identity of GM-CSF with almost complete sequence coverage (Fig. 3).

### rhGM-CSF Bioactivity

**Figure 4:**
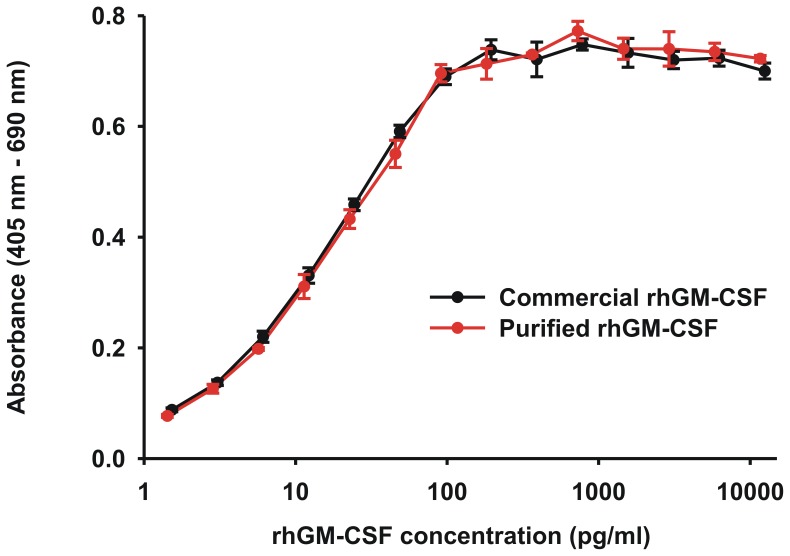
Purified rhGM-CSF demonstrates bioactivity. Shown is the proliferation of TF-1 cells after incubation for 4 days in the presence of purified rhGM-CSF. For comparison, a commercial source of rhGM-CSF was also analyzed. TF-1 cells require the presence of GM-CSF for survival and therefore their proliferation with the addition of GM-CSF, as measured using the WST-1 reagent, is a measure of GM-CSF bioactivity. Absorbance values are proportional to the number of viable cells. Data are the mean ±SD of triplicate measurements.

To confirm the bioactivity of the refolded, purified rhGM-CSF, a human leukemia cell, TF-1, that was dependent on GM-CSF for its survival and proliferation, was washed extensively and 1000 cells were placed into 96 wells with a titration of refolded rhGM-CSF in triplicate. Four days later the metabolic activity of the surviving TF-1 cells was assessed by a WST assay according to the manufacturer’s instructions. In parallel, a titration of a standard commercial preparation of rhGM-CSF obtained from ImmunoTools was performed. The titrations of purified rhGM-CSF or the commercial source started with concentration of 1.28 ng/ml of each. As can be seen in Fig. 4, the purified rhGM-CSF promoted 50% maximal growth of the TF-1 cells at a concentration of 12.5 pg/ml (4.0×10^9^ U/mg) with the commercial rhGM-CSF having comparable activity at 11.4 pg/ml (4.4×10^9^ U/mg).

## Discussion

We had previously developed a protocol that efficiently allowed for the refolding of Fab fragments of antibodies from inclusion bodies [19]. Therefore, because rhGM-CSF forms inclusion bodies in *E. coli,* we adapted our refolding protocol for its purification. Our protocol is based on a combination of strategies for refolding of inclusion body proteins. For a review of isolation and purification of proteins from inclusion bodies see Vallejo and Rinas [12]. After isolation of the inclusion bodies from the *E. coli* cell lysate, the first step in the rhGM-CSF refolding procedure is the solubilization of the inclusion bodies. (Our previous work refolding Fab fragments found that extensive washing of the inclusion body pellet did not improve the refolding protein yields, so we therefore do not include a wash step.) The conditions we used to resuspend the rhGM-CSF were harsh, however they were effective at solubilizing the protein and reducing non-native, inter- and intramolecular disulfide bonds. As well, although expensive, we found that use of a high grade of GuHCl was necessary to achieve efficient refolding.

Once solubilized, the protein was dialyzed against decreasing amounts of GuHCl. It is common to use dilution to reduce the amount of GuHCl during protein refolding, however this results in large volumes of dilute protein which is difficult to scale-up. Dialysis is more compatible with higher concentrations of protein and we found that it was effective for refolding both Fabs and rhGM-CSF. To aid in the rhGM-CSF refolding, we first reduced the disulfide bonds using DTT. hGM-CSF contains 4 cysteine residues forming 2 disulphide bonds that likely formed non-native, inter- and intramolecular disulfide bonds during inclusion body formation [10]. To aid in the generation of the correct disulphide bond configuration, we included reduced and oxidized glutathione in the dialysis buffer with the reduced form in excess. Arginine was also included in the dialysis buffer to help prevent aggregation of partially folded intermediates. The exact mechanism by which L-arginine prevents the aggregation of folding intermediates remains unresolved but may be due to its effects on the structure of water or its interactions with the protein itself [16], [17].

The rhGM-CSF was purified using a nickel-chelating resin via the 8×His tag engineered on its C-terminus. Immediately after elution from the nickel column, we dialyzed the rhGM-CSF against a low salt containing buffer. In doing so, any partially folded protein precipitated and was removed during a subsequent centrifugation step. In cloning the rhGM-CSF into the expression vector, the additional amino acids, LE +8×His tag, were added to the C-terminus of the protein. Crystallographic analysis of rhGM-CSF [9] indicates that the C-terminus points away from the molecule and into the solvent decreasing the likelihood that the additional residues on the C-terminus would have an effect on rhGM-CSF function. However, to verify this we analyzed the bioactivity of the purified rhGM-CSF. The resultant protein was bioactive and was able to promote 50% maximal growth of TF-1 cells at a concentration of 12.5 pg/ml. The specific bioactivity was 4.0×10^9^ U/mg and very similar to that of a commercial source of rhGM-CSF.

rhGM-CSF has been refolded from inclusion bodies for many years. Early attempts similarly used chaotropic and reducing agents to solubilize the rhGM-CSF from inclusion bodies with dialysis into neutral Tris buffer [23] or chaotropic agents combined with extremes of pH [11], for example. However, the final yields of rhGM-CSF in these instances were significantly lower (∼0.4–0.5 mg per lire of *E. coli*). Likely it was the lack of addition of L-arginine and reduced and oxidized thiols that may have lowered the final yields of rhGM-CSF in these refolding protocols. Indeed, it has been demonstrated that enabling proper disulphide bond formation through the use of reduced and oxidized thiols and the addition of L-arginine to prevent aggregation are key to improving yields of purified, active protein [18], [24]. An alternative approach to producing rhGM-CSF is to prevent the formation of inclusion bodies in the first place. Expression systems that allow for the recovery of soluble and purified rhGM-CSF from the periplasmic space of *E. coli* have been successful, although the final yields can be significantly lower (∼500 µg/ml) [25]. Alternatively, expression of proteins of interest as fusion proteins has been shown to aid in the production of soluble, active protein if the target is normally susceptible to inclusion body formation [26]. rhGM-CSF has successfully been fused to either Thioredoxin (TRX) or an intein resulting in the production of high yields of purified rhGM-CSF [27], [28]. Although in these instances, enzymatic cleavage and separation steps are required to remove the fusion partner, these methods are advantageous as they are not hampered by inclusion body formation.

### Conclusions

Once inclusion bodies are formed, it can be difficult to refold the protein of interest into an active form. Here we present an easy, straightforward and efficient protocol for the purification of rhGM-CSF from inclusion bodies that was also successfully used in the refolding and purification of antibody Fab fragments. It involves the expression of the protein of interest in *E. coli*, solubilization from inclusion bodies, refolding by dialysis, and purification on a nickel-chelating resin via a C-terminal His-tag. This protocol does not require extensive experience in protein purification nor elaborate chromatography equipment. Using this protocol we routinely generate approximately 7 mg of bioactive rhGM-CSF per litre of cell culture.
